# Genome-wide association mapping for heat shock tolerance in *Mercenaria mercenaria* through SNP microarray analysis

**DOI:** 10.1186/s12864-025-11689-5

**Published:** 2025-05-30

**Authors:** Huiping Yang, Denis Grouzdev, Zhenwei Wang, Jayme C Yee, Yangqing Zeng, Leslie Sturmer, Bassem Allam

**Affiliations:** 1 School of Forest, Fisheries, and Geomatics Sciences, Institute of Food and Agricultural Sciences, University of Florida, 7922 NW 71st Street, Gainesville, FL 32653 USA; 2 School of Marine and Atmospheric Sciences, Stony Brook University, Stony Brook, NY 11794-5000 USA; 3 Haskin Shellfish Research Laboratory, Department of Marine & Coastal Sciences, Rutgers University, 6959 Miller Avenue, Port Norris, NJ 08349 USA; 4 Nature Coast Biological Station, University of Florida, Cedar Key, Florida, 32625 USA

**Keywords:** Northern quahogs, Heat tolerance, GWAS, SNP microarray, *Mercenaria mercenaria*, Hard clams

## Abstract

**Background:**

The northern quahog *Mercenaria mercenaria* is a major aquaculture species on the US East Coast, and heat resistance is the most sought trait for aquaculture. This study aimed to establish a genome-wide association for heat tolerance using a 66K SNP array for *M. mercenaria*. Quahogs from three farms were combined for a heat challenge at 1 °C per day from 24 °C to 35 °C and stay for two days (Phase I), decreasing to 27 °C in 24 h, to 24 °C in another 24 h, and maintaining at 24 °C (Phase II) until no one dead within 48 h at 24 °C (Phase III). Dead and live quahogs were sampled for genotyping using the SNP array.

**Results:**

During the heat challenge, different mortalities among the quahogs from the three farms were identified at 38, 46, and 55% at Phase I, and 36, 30, and 29% at Phase II. For the survivors (Phase III), no changes were found in body weight before and after the heat shock challenges (*p* < 0.265). The PCA analyses of SNP frequencies indicated significant genetic differences associated with quahog survival under heat stress across the different farms. The heritability of the heat tolerance was 0.680 ± 0.063. GWAS analysis indicated that one SNP exhibited a significant association with the time-to-death trait on chromosome 7 (*p* = 1.98 × 10^− 5^). More significant SNPs (*p* < 10^− 3.5^) were inside genes that have been reported to function in heat tolerance such as *serine/threonine-protein kinase 31* and *carbohydrate sulfotransferase 11*, and some genes found within 50 K bp far from SNP sites have a relationship with heat tolerance such as *toll-like receptors 4* and *6* (*TLRs 4* and *TLRs 6*), *uracil-DNA glycosylase*, and a *disintegrin and metalloproteinase with thrombospondin motifs gon-1* (*ADAMT*s).

**Conclusion:**

The fastStructure analysis revealed the proportions of different ancestral components within the quahogs from different farming stocks, highlighting that the genetic factors may contribute to their varying survival rates under heat stress. The associated genes have potential roles in immune response, cellular stress, and tissue repair. The findings highlighted the power of high-throughput approaches for the identification of superior quahog genotypes for further breeding.

## Background

Water temperature is one of the essential factors in aquatic ecosystems to determine the growth, metabolic rates, and survival of aquatic organisms [1]. In marine ecosystems, sea surface temperature increased at an average rate of 0.14 °F per decade from 1901 to 2020 and was recorded as the warmest in 2023 (the US Environmental Protection Agency data). The increase in sea surface temperature would affect species distribution, migration, and breeding patterns, change the circulation pattern and nutrient supply in coastal regions, and impact coastal aquaculture industries [2, 3]. Furthermore, other correlated effects, such as high occurrence of coastal flooding, ocean acidification, and sea level rise, also pose severe threats to coastal marine aquaculture [4]. Globally, coastal economies rely on fisheries and aquaculture [5]. Molluscan bivalves are particularly favorable aquaculture species because of their filter-feeding lifestyle, which has beneficial impacts on the environment. Bivalve aquaculture requires no supplemental feeding and can clean the water column for nutrient recycling and create substrates for other species [6]. Over the past three decades, molluscan aquaculture has more than doubled, accounting for about 56.3% of the global marine aquaculture production [7]. Aquaculture of molluscan bivalves provides the opportunity for selective breeding to improve resilience to environmental stressors [8, 9].

The northern quahog, *Mercenaria mercenaria* (also called hard clam), is a native edible intertidal molluscan bivalve along the western Atlantic coast from the Gulf of St. Lawrence to Florida [10]. Aquaculture of *M. mercenaria* has been developed since the 1970s, and the species has been introduced to other countries for aquaculture [11], now representing a $57.5 million industry (sales value) in 12 East Coast states in the US, with Florida as the top producer [12]. This quahog aquaculture industry is entirely reliant on commercial hatchery seed, and thus, the production of high-quality seed able to resist biotic and abiotic stresses through genetic breeding is a feasible approach to improve aquaculture. In Florida, the subtropical temperatures provide a long growing season for quahog aquaculture, meanwhile, the high temperature in summer months may also exceed the optimum temperature range since Florida is the southernmost limit of the natural distribution of northern quahogs. In situ water monitoring in Cedar Key, which represents the main area of hard clam aquaculture in Florida, showed that the water temperature reached 34.09℃ in August of 2024 (https://shellfish.ifas.ufl.edu/water-quality-monitoring/), and examination of historical water temperature data indicated that the high-temperature records have an increasing trend. Overall, the summer heat is currently the biggest challenge for Florida’s clam aquaculture industry and has been widely recognized by the clam industry because the market-sized clams are often vulnerable and suffer high mortalities.

Previous quahog breeding efforts included mass selection and family selection in the 1990s, and positive selection on growth rate in juveniles and adults was obtained in only one generation [13]. Outcrossing among broodstock from different hatcheries did not show heterotic effects, but a strong correlation between the performance of seed and adults was identified, suggesting that effective selection could be conducted at juvenile age [14]. Additionally, efforts were made on triploid production for fast growth and overcoming summer heat resilience [15–18], and hybridization of *M. mercenaria* with a sister southern species *Mercenaria campechiensis* was performed in an attempt to enhance fast growth, heat tolerance, and longer shelf-life [19, 20]. Despite these tremendous efforts, no established genetic strains/lines have been reported or used for commercial seed production.

The integration of genomic tools, particularly SNP arrays, into aquaculture breeding programs has revolutionized the major molluscan aquaculture species by enabling detailed genetic analyses and improving breeding strategies [21–24] with publicly accessible breeding platforms [25]. For the northern quahogs, a significant advancement was the creation of a 66K SNP array, specifically designed to facilitate comprehensive genetic studies and support targeted selective breeding efforts to enhance clam resilience and productivity [26]. This study aimed to establish the genome-wide association of heat tolerance with genotypes through the newly developed 66KSNP array for *M. mercenaria*. Results from this study are expected to lay the foundation for further genomic selection for heat tolerance of quahogs, speeding up and supporting the large-scale clam industry in Florida.

## Materials and methods

### Animals

Adults *M. mercenaria* (12–14 months old) were collected from three farms in Cedar Key, Florida, with one whole bag from each farm (1000–1200 clams). The clam seeds of each farm were originally sourced from different hatcheries. After purchase, the quahogs were cleaned at the farms using fresh water to remove sediment before being transported to the laboratory in a cooler (1 h driving). After arriving at the laboratory, all quahogs were maintained in an indoor recirculating artificial seawater system (total volume = 2,400 L) following our standardized protocol [27]. The recirculating system is equipped with a bead filter (Endurance 4000, Aquaculture Systems Technologies, Baton Rouge, LA, USA) and an 80-W UV light (E80S SMART UV high-output system, Pentair Aquatics, Apopka, FL, USA), and water parameters were maintained at 24 ± 0.55 °C, 25 ppt salinity, and pH = 8.35. Quahogs were fed daily with fresh microalgae, including *Tetraselmis* spp., *Isochrysis* spp., *Thalassiosira* spp., and *Chaetoceros* spp. supplemented with commercial algal paste (Instant algae, Reed Mariculture, San Jose, CA, USA) at a final 50,000 cells/ml. Water quality was checked daily using a YSI probe (Professional Plus, YSI Incorporated, Yellow Springs, OH, USA) for salinity, temperature, oxygen, and pH, and a saltwater combination kit (AQ-4, Lamotte, MD, USA) for ammonia (< 0.01 mg/L), nitrite (< 0.01 mg/L), and nitrate levels (< 10 mg/L).

From each farm, 400 quahogs were randomly selected for use in this study. All quahogs were labeled with a permanent marker, and shell metrics were measured (height, length, and width in mm) and body weight (grams). Following a 2-week acclimation, a total of 1050 quahogs (350 from each farm) were challenged with heat, meanwhile, a total of 150 quahogs (50 from each farm) were kept at 24 °C as controls.

### Heat challenge

**Fig. 1:**
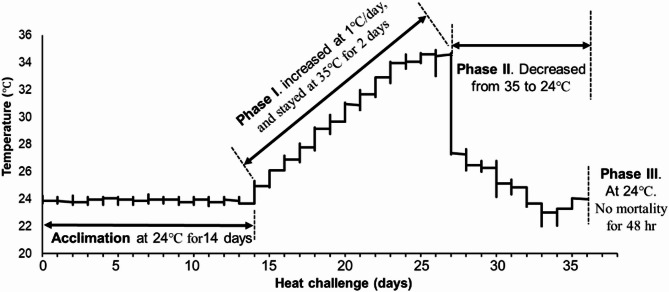
The temperature profile of the heat challenge of northern quahogs *Mercenaria mercenaria*

Based on our pilot experimental results (not shown here) and a literature review of heat tolerance of adult clams, the heat shock challenge was applied by increasing water temperature at 1 °C per day from 24 °C to a final temperature of 35 °C using a heat pump with a temperature controller (Model DC24S, Aqualogic, San Diego, CA, USA). When the total dead quahog numbers reached ∼ 50% of the total initial clam number, the temperature decreased from 35 °C to 27 °C within 24 h and gradually to 24 °C within another 24 h. For easy reading, the heat challenge was separated into three phases (Fig. 1): **Phase I** (13 days)– temperature increased from 24 °C to 35 °C and stayed at 35 °C; **Phase II** (9 days)– temperature decreased from 35 °C to 24 °C with additional clam mortality observed, and **Phase III**– temperature was maintained at 24 °C and no quahog mortality was found in two days. Two HOBO pendant temperature loggers (UA-001–64, Onset, MA, USA) were submerged in the tank to record the temperature changes. No feeding was conducted after the temperature reached 28 °C because a temperature range of 16–27 °C is considered the optimum for feeding and growth, and the siphon pumping rate of adult northern quahogs declined sharply above 27 °C [28]. Water quality was monitored twice a day.

### Phenotype record

Survival was monitored every two hours throughout the heat challenge period (Phases I and II). The dead quahogs were defined as shells opened without response after being touched. Dead quahogs were picked up by squeezing shells together to prevent decayed tissues from dropping into the tank. The date, time, label number of each quahog, and body weight were recorded; each quahog was photographed, and pieces of adductor muscle or other tissue (if adductor muscle is not available) were fixed in 96% ethanol in a 2-ml tube. After replacing the fixative (96% ethanol) twice on days 1 and 3, the fixed samples were placed in a refrigerator at 4 °C for storage. For quahog survivors from the heat challenge (Phase III), 1–2 ml of hemolymph was extracted from each quahog in a 2 ml microcentrifuge tube following our previously described method [29]. The hemolymph was immediately centrifuged for 10 min at 4000 rpm at 4 °C. The supernatant was removed and discarded by pipetting without disturbing the cell pellet, and 1 ml of 96% ethanol was added to the tube to fix the hemocyte pellet. Fixed samples were also placed in a refrigerator at 4 °C for storage.

### Genotyping of samples

A portion of the samples during the heat challenge was genotyped. The quahogs that died earliest (*n* = 330) in Phase I were categorized as “dead” under the dichotomous phenotype, and the quahogs that died latest (*n* = 144) in Phase II and the survivors (*n* = 231) in Phase III were categorized as “survived”. Genotyping of samples was performed on the previously described SNP microarray chip [26]. Fixed samples were shipped to the Center for Aquaculture Technologies (CAT, https://aquatechcenter.com, San Diego, CA, USA) for this service. The total genomic DNA was extracted from sample sources (adductor muscle or hemocytes) using a magnetic bead-based extraction protocol. Briefly, fixed tissues (∼ 10–15 mg) were subsampled and processed using Mag-Bind blood and tissue DNA kits (Omega BioTek, Norcross, GA, USA) according to the manufacturer’s guidelines. Automated processing and liquid handling steps associated with the extraction protocol were performed using PurePrep 96 units (Molgen, San Diego, CA, USA) according to kit and instrument-specific guidelines. Extracted gDNAs were assessed for yields and quality by spectrophotometry (Nanodrop, ThermoFisher) and 2% agarose gels targeting a minimum of 35 µl at > 20 ng/µl of largely intact DNA (minimum 5KB). Genotyping was performed at Neogen (Lincoln, NE, USA) on the Axiom 384HT arrays (Axiom HD Array [60 K] Clam) following protocols outlined in the Axiom Assay 384HT Array Format Automated Workflow User Guide.

The raw genotyping data in CEL format was downloaded from the CAT website after the service and genotype annotation was analyzed using the Axiom software (v 5.4, Thermo Fisher) following the Best Practices Workflow with recommended threshold settings (DishQC ≥ 0.82, QC call rate ≥ 97%). Genotypes were exported in PLINK format (along with summary QC statistics) for further filtering and use in downstream analyses. Further quality control measures were implemented using PLINK v 1.90 [30]. To filter out low-quality data. Genotypes were filtered to remove individuals with more than 5% missing data (--mind 0.05), variants with a minor allele frequency MAF lower than 5% (--maf 0.05), and variants with more than 5% missing genotypes (--geno 0.05). Additionally, duplicate individuals identified in the previous step were deduplicated. These filtering steps resulted in a final dataset comprising 38,783 variants across 633 clams.

### Genetic diversity and structure analysis

Principal component analysis (PCA) was performed using PLINK v1.90 [30]. Genetic diversity indices, including observed heterozygosity (Hobs), expected heterozygosity (Hexp), and inbreeding coefficient (Fis), were calculated using SambaR v 1.10 [31]. Population differentiation was assessed by calculating pairwise fixation index (Fst) values using the ‘stamppFst’ function in the R package StAMPP v1.6.3 [32]. Structure analysis was performed with fastSTRUCTURE v 1.0 [33] using a logistic prior and cross-validation (cv = 10) over values of K from 1 to 10 to estimate the most likely number of genetic clusters. The optimal number of clusters (K) was determined using the MedMeaK, MaxMeaK, MedMedK, and MaxMedK metrics [34] via the StructureSelector web tool (https://lmme.ac.cn/StructureSelector/) [35]. Cluster partitions were then visualized using Pong v 1.5 [36].

### Genome-wide association analysis

After the QC and imputation, the data were used to conduct the Genome-Wide Association Studies (GWAS). The traits analyzed included clam survival and time to death (TTD). Survival status (dead or survivored) was used as the binary trait to represent heat tolerance. The GWAS was performed using the ASRgwas R package and the following linear mixed model [37, 38]:

$$y = X\beta + Zg + S\tau + \varepsilon $$

where β is a vector of fixed effects from both environment factors and population structure, g is the model for the genetic background of each line as a random effect, τ models the additive SNP effects as a fixed effect, and y is the observed trait.

GWAS accounting for population structure and relatedness was conducted to account for the phenotypic covariance due to genetic relatedness (Q + K model or MLM). The output of GWAS was visualized as a Manhattan plot and a quantile-quantile (QQ) plot using the QQ-man package on R [39]. In the Manhattan plot, the position on the chromosome was on the x-axis, and the -log10 of the *P*-value of the tested SNPs was on the y-axis. To avoid artificial associations in the genetic study, the kinship between individuals was considered during the GWAS analysis. This step helps to control the fact that relatives share more genetic markers simply because they are related, not necessarily because those markers are associated with the heat tolerance trait. The chromosome suggestive threshold (-log10 (0.05/ (Number of markers/Number of chromosomes)) was applied to selected markers associated with heat tolerance [40, 41]. The Bonferroni correction value was set to 0.05 to adjust the number of independent tests performed. Based on our literature review, the distance for the upstream and downstream gene identification has been reported to be small (2 kb) or large (up to 500 kb) cutoff thresholds, depending on specific research questions, species, and data availability. In this study, the candidate genes located 50-kbp upstream and downstream of the selected marker position were identified according to the hard clam reference genome [42] (https://www.ncbi.nlm.nih.gov/datasets/genome/GCF_021730395.1/).

### SNP-based heritability

The SNP-based heritability was estimated using the variance components approach. The variance components were estimated on ASReml via the residual maximum likelihood (REML) method [43]. The REML approach is widely used to estimate variance components and thus heritability [44]. REML is based on large sample theory under the assumption that the parameter estimates are asymptotically multivariate normally distributed. The covariance matrix is given by the inverse of the information matrix. In practical terms, considering a model with two random effects, one accounting for the variability explained by the genomic component and a residual term addressing the unexplained variability, ASReml R calculates the heritability by summing the first and second variance components (the additive and residual variances). Then, heritability was calculated as the ratio of the variance component associated with the genomic component (additive variance) and the sum of the corresponding variance components of the genomic and residual terms (phenotypic variance).

### Statistical analysis

The phenotypic data analysis was performed by using JMP^®^ Pro (Version 17, SAS Institute Inc., Cary, NC, 1989–2023). The shell metrics and body weight of clams were expressed as Mean ± Standard Deviation (SD), and survival during the heat shock challenge was expressed as a percentage. Survival-time analysis was used to test the quahogs’ survival against the time survival (in days). The comparison of parameters among the three farming populations was conducted by ANOVA analysis. The Arcsine square root transformation was applied to percentage data for normality. The significance was set when the *p*-value was < 0.05.

## Results

### Survival of the clams during heat shock challenge

The quahog shell metrics and body weights (mean ± SD) from each farm for the heat challenge are listed in Table 1. The quahogs from farm 1 were much smaller than those from the other two farms in body length (*p* < 0.0001), body height (*p* < 0.0001), body width (*p* < 0.0001), and body weight (*p* < 0.0001). Between farms 2 and 3, the difference was found in body length only (*p* = 0.007), but not in body height (*p* = 0.943), body width (*p* = 0.779), and body weight (*p* = 0.380).

**Table 1:** Shell metrics (mm) of *Mercenaria mercenaria* per farm and corresponding standard deviation for the genotypes used for heat challenge and body weight (g) changes during the challenge period

Farm	Body length (mm)	Body height (mm)	Body width (mm)	Body Weight (g)	Body weight of Survivors (g)	
					Before heat	After heat
1 (CK)	48.71 ± 4.41	40.11 ± 3.53	25.78 ± 2.55	34.90 ± 9.04	35.65 ± 8.80	35.81 ± 8.80
2 (SC)	51.95 ± 3.47	43.57 ± 3.06	27.57 ± 2.20	42.94 ± 7.99	42.33 ± 7.34	42.52 ± 7.36
3 (UF)	51.06 ± 3.22	43.62 ± 2.59	27.45 ± 1.96	42.11 ± 6.29	40.88 ± 4.97	40.30 ± 5.39

**Fig. 2:**
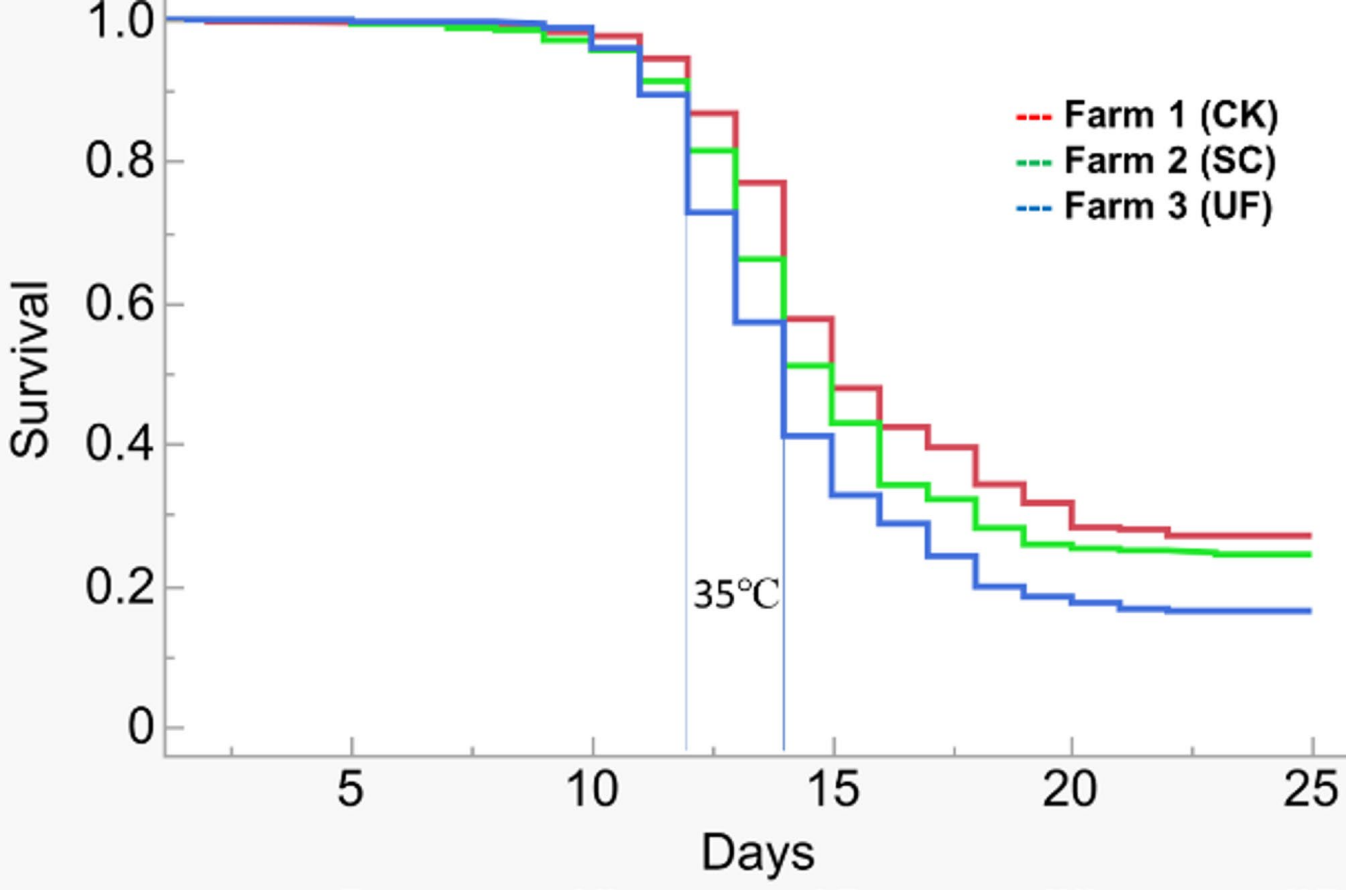
The survival plot of northern quahogs *Mercenaria mercenaria* from three farms in response to a heat challenge (from 24℃ to 35℃ at 1℃/day for 10 days, stayed at 35℃ for 3 days, and decreased to 27℃ for 1 day, and to 24℃ for 9 days when no mortality was observed for two days)

During the acclimation period, mortality was observed (Table 2), but was not considered the response to the heat challenge. Survival-time analysis indicated the survivorship of quahogs from these three farms (Table 2) was different in response to the heat challenge (Fig. 2, *P* < 0.0001 log-rank and Wilcoxon tests), and the mortality/survival percentage at each Phase of the heat challenge was listed in Table 2. During the heat shock period, the sampled dead quahogs were all shell open, and the body weight values were significantly lower than those before starting the heat shock (data not shown). For the survivors, no changes were found in body weight before and after the heat shock challenges (Table 1, *p* < 0.265).

**Table 2:** Mortality and survival of the *Mercenaria mercenaria* during the heat shock challenge

	Acclimation (mortality)	Survived quahogs (%)			
		Quahog#	Phase I (mortality)	Phase II (mortality)	Phase III (Survival)
**Heat Challenge**	24℃		From 24 to 35℃ @ 1℃/day and at 35℃	From 35℃ to 26℃	At 24℃ (10 days after heat shock)
Farm 1 (CK)	15	348	131 (38%)	124 (36%)	93 (26%)
Farm 2 (SC)	19	347	159 (46%)	104 (30%)	84 (24%)
Farm 3 (UF)	3	350	193 (55%)	100 (29%)	57 (16%)
**TOTAL**		**1045**	**483**	**328**	**223**
Farm 1 (CK)	**Quahog # for genotyping**		88	56	92
Farm 2 (SC)			90	44	82
Farm 3 (UF)			152	44	57
**TOTAL**			**330**	**144**	**231**

Estimation of the parametric survival fit model showed that the quahog survival was highly correlated with the heat shock temperature at each phase of the challenge, and the quahog shell metrics and body weight (effect likelihood ratio tests *p* ≤ 0.0012).

### Genetic diversity and structure analysis

**Fig. 3:**
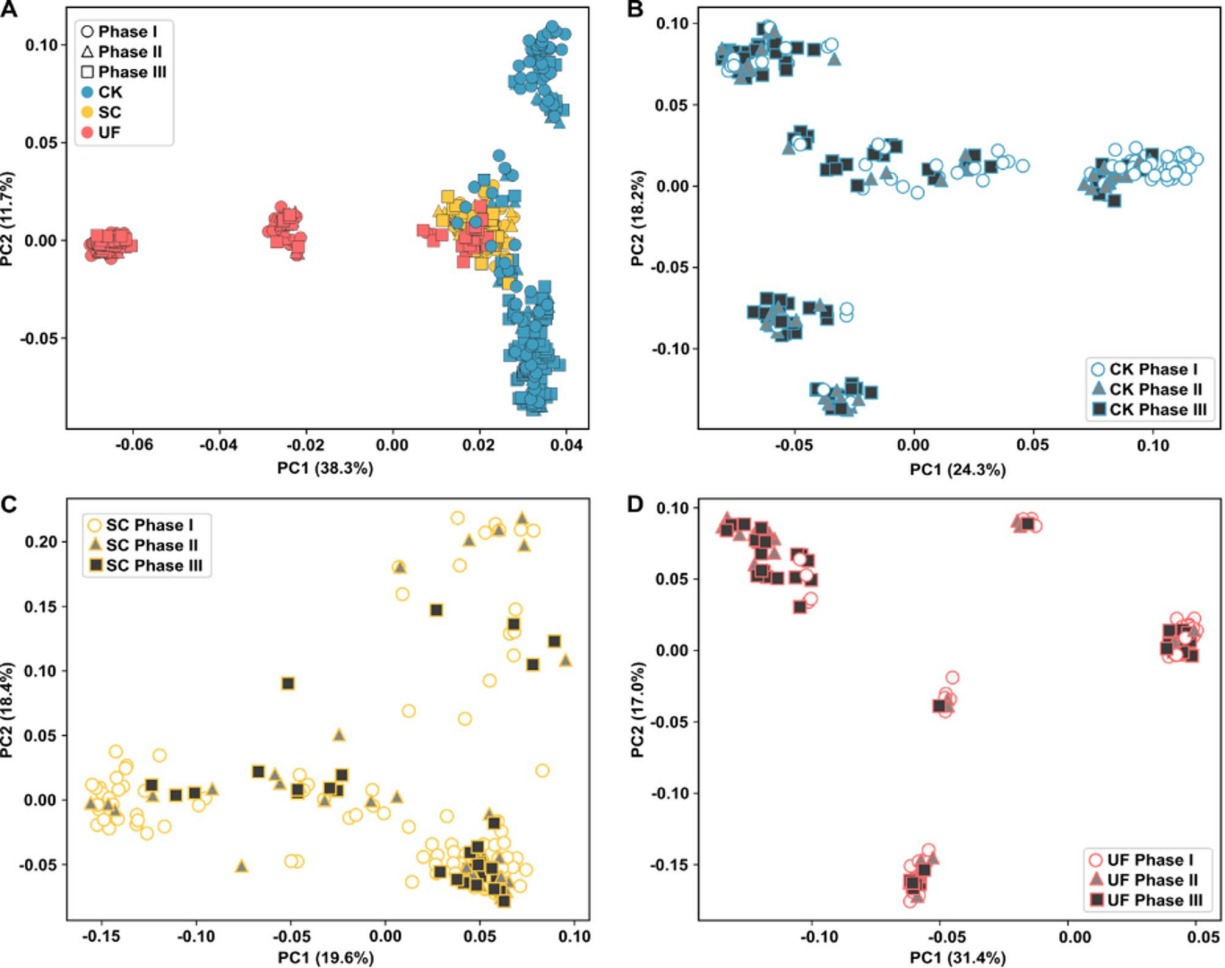
Principal component analysis of *Mercenaria mercenaria* derived from different farms and collected during different phases of the heat shock challenges. (**A**) PCA results by combining all samples, illustrating genetic variability across three farms: Farm 1 (CK), Farm 2 (SC), and Farm 3 (UF), each represented by different colors. The phases within each farm are indicated by different shapes: circles for Phase I, triangles for Phase II, and squares for Phase III, demonstrating the genetic differentiation among phases and farms. (**B-D**) PCA results for each farm individually: (**B**) for Farm 1 (CK), (**C**) for Farm 2 (SC), and (**D**) for Farm 3 (UF). The phases are distinguished by color fillings: white for Phase I, grey for Phase II, and black for Phase III

The PCA results provided insights into the genetic structure of *M. mercenaria* derived from different farms and phases of heat shock challenge (Fig. 3). The combined analysis of all samples (Fig. 3A) demonstrated a distinct pattern of genetic segregation based on the farm of origin. The PCA revealed that clams from Farm 3 (UF) are split into three distinct clusters (Fig. 3A). One of these clusters is positioned at a considerable distance from the others, which suggests a unique genetic composition. In contrast, the clams from Farm 2 (SC) are clustered tightly together, showing less genetic diversity, and they are mixed with the last UF cluster. Quahogs from Farm 1 (CK) also exhibit three separate clusters. One of these clusters overlaps with the SC and UF animals, indicating that some CK clams share genetic similarities with these other groups.

The results of the PCA analyses indicated significant genetic differences associated with clam survival under heat stress across the different farms. In Farm 1 (CK), some clusters predominantly consist of clams that died in Phase I, which suggests a lower resilience to heat stress (Fig. 3B). A similar pattern is observed in Farm 3 (UF), where specific clusters correlate with higher mortality in Phase I (Fig. 3D). In contrast, Farm 2 (SC) shows fewer distinctions between the phases (Fig. 3C).

**Fig. 4:**
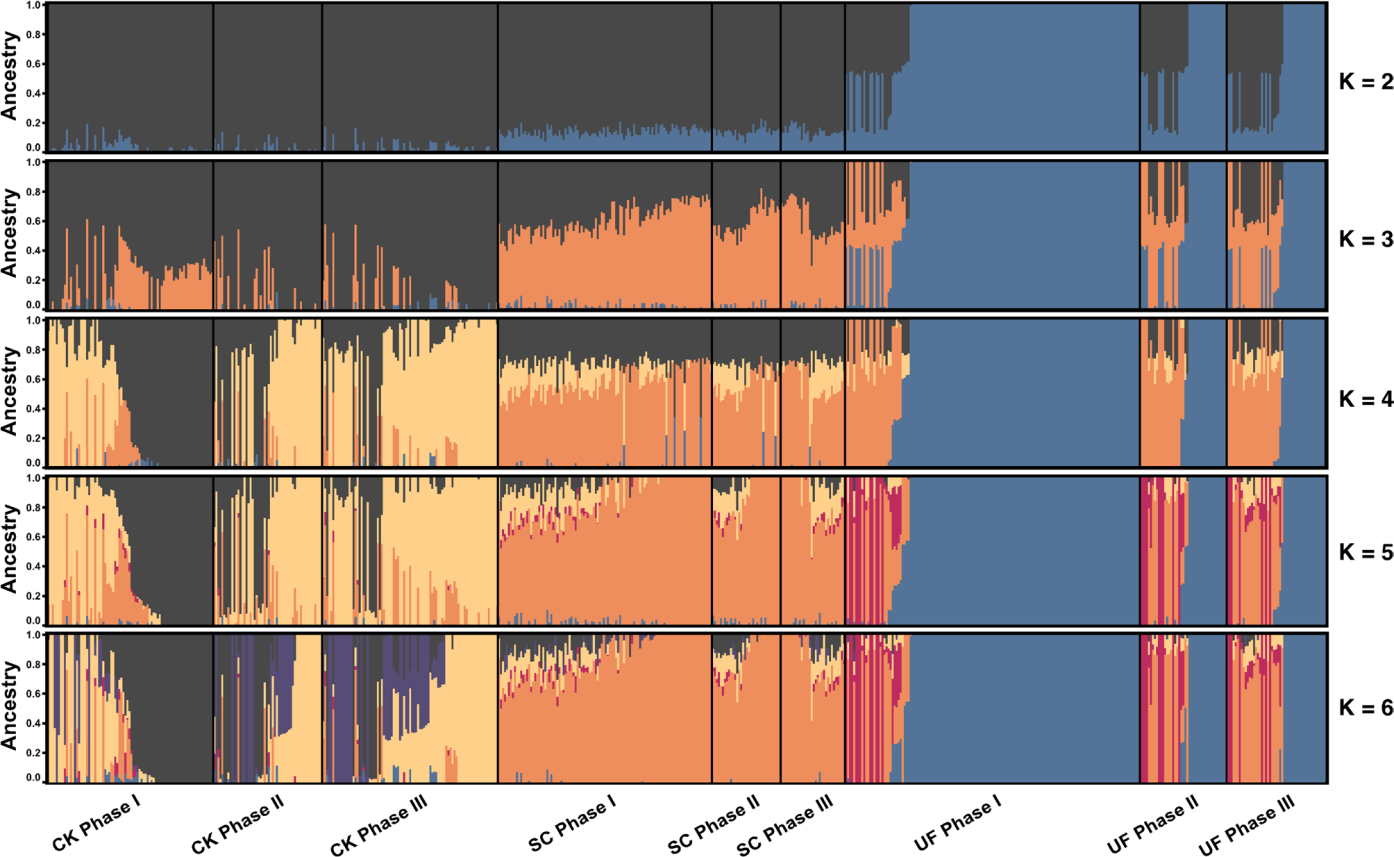
Population structure analysis of ***Mercenaria mercenaria*using FastStructure software for different farms and phases of heat shock challenge.** This Figure shows the proportional ancestry of clams from three different farms. Each row represents a different number of assumed genetic clusters (K), ranging from K = 2 to K = 6. Within each farm, the clams are grouped into three phases of the heat shock challenge: Phase I, Phase II, and Phase III, indicated by the panel divisions. The color gradients represent different ancestral components identified by FastStructure analysis

The population structure analysis conducted using fastStructure further supported the results of the PCA by providing a deeper insight into the genetic composition of *M. mercenaria* across farms and heat shock phases (Fig. 4). This analysis revealed the proportions of different ancestral components within the clams, highlighting the genetic factors that could contribute to their varying survival rates under heat stress. To further refine the understanding of the genetic structure and ensure the robustness of our analysis, the optimal number of genetic clusters (K) using the MedMeak, MaxMeak, MedMedK, and MaxMedK metrics was assessed. Each of these metrics consistently indicated that K = 4 was the optimal number of genetic clusters.

Significant genetic heterogeneity was observed in clams from Farm 1 (CK). It is noteworthy that one of the ancestral components (dark grey, K = 4) was predominant in clams that succumbed in Phase I, comprising 58.7% of their genetic makeup. This component comprised only 20.5% of the genetic makeup of the survivors of Phase III (Fig. 4). In contrast, clams from Farm 2 (SC) exhibited a more uniform genetic structure. The third farm (UF) exhibited a distinctive pattern. The FastStructure analysis identified a distinct ancestral component (dark blue, K = 4) that predominated in the genetic makeup of clams that died in Phase I, accounting for 85.0% of their ancestry, compared to 50.7% in those that survived to Phase III (Fig. 4). This distinctive ancestry in UF is particularly noteworthy, as it indicates the potential presence of specific genetic traits that may be particularly vulnerable to heat stress, thereby differentiating these clams from those observed in other farms.

The results of genetic diversity and inbreeding coefficients across farms and phases of the experiment (Table 3) were consistent with the results of the PCA and FastStructure analyses.

**Table 3:** Genetic diversity and inbreeding coefficients of *Mercenaria mercenaria* throughout the heat challenge experiment

Farm	Group	Fis	Hobs	Hexp
	Phase I	-0.001	0.391	0.389
1 (CK)	Phase II	-0.009	0.392	0.387
	Phase III	0.002	0.386	0.385
	Phase I	0.037	0.391	0.405
2 (SC)	Phase II	0.039	0.390	0.405
	Phase III	0.020	0.396	0.404
	Phase I	-0.056	0.375	0.332
3 (UF)	Phase II	-0.002	0.377	0.370
	Phase III	0.011	0.380	0.379

For quahogs from Farm 1 (CK), the Fis values are close to zero in phases I and II, indicating minimal inbreeding, with a slight increase in phase III (Fis = 0.002), consistent with a slight decrease in genetic diversity (Hobs and Hexp). For quahogs from farm 2 (SC), Fis values are slightly positive in all phases, indicating some inbreeding, but the observed and expected heterozygosity (Hobs and Hexp) remained relatively stable and higher than those in CK and UF, especially in Phase III. In quahogs from Farm 3 (UF), the significant negative Fis in Phase I (-0.056) indicates outbreeding or a complex genetic structure, possibly reflecting a diverse genetic background that has not conferred heat resistance. By Phase III, the Fis has shifted to a positive value (0.011), highlighting genetic consolidation among the survivors. The increases in both observed and expected heterozygosity from Phase I (Hobs = 0.375, Hexp = 0.332) to Phase III (Hobs = 0.380, Hexp = 0.379) were consistent with the FastStructure results, where clams that perished in Phase I predominantly had a strong ancestral component (Fig. 4), which was less prevalent in those that survived to Phase III.

**Fig. 5:**
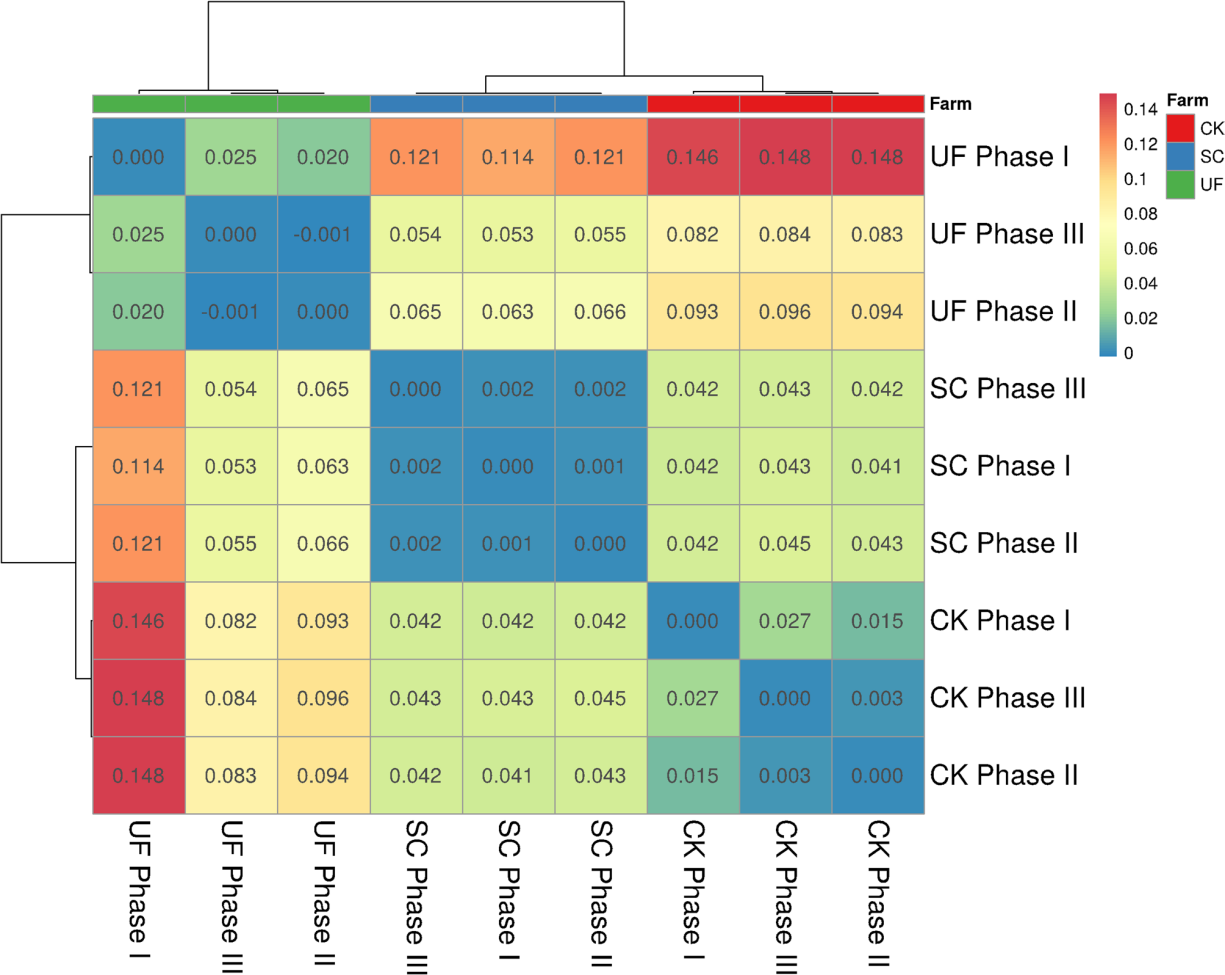
Heatmap of pairwise *F*_*ST*_ values summarizing genetic differentiation between different groups and farms. Each cell represents the *F*_*ST*_ value for the intersection of the respective clam’s group on the x and y axes

The Fst analysis (Fig. 5) reveals that clams from Farm 2 (SC) show minimal genetic differentiation across phases, indicating a relatively uniform genetic background.

In contrast, quahogs from Farm 1 (CK) and Farm 3 (UF) displayed notable genetic differentiation between phases. Specifically, clams that perished in Phase I exhibit genetic divergence from those that succumbed in Phase II and from survivors in Phase III, indicating selective pressures that favor specific genetic profiles for heat resilience. These findings corroborate those of the PCA and FastStructure, underscoring the close association between genetic differentiation in CK and UF clams and survival outcomes.

### Genome-wide analysis

**Fig. 6:**
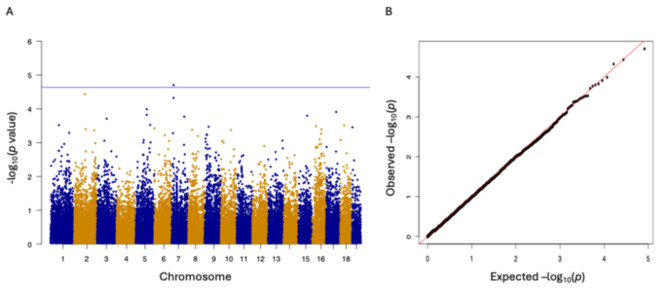
GWAS of heat tolerance in the northern quahog *Mercenaria mercenaria*. **A**: The Manhattan plot of the time to death trait. **B**: QQ plot of time to death trait. The blue lines in A represents the threshold for Bonferroni correction.

The dataset of 38,783 SNPs was used to assess the heat tolerance of northern quahogs. The heritability of the heat tolerance traits was determined as 0.680 ± 0.063. As shown in Fig. 6A, one SNP in chromosome 7 exhibited a significant association with the time-to-death trait of northern quahogs. More SNPs were also selected with a *p*-value smaller than 10^− 3.5^. Some outlier SNPs were located inside genes that have been reported to be involved in heat tolerance, such as *serine/threonine-protein kinase 31* and *carbohydrate sulfotransferase 11*. Some SNP sites were within 50 K bp far from genes that have a relationship with heat tolerance, such as *toll-like receptors 4 and 6* (*TLRs 4* and *TLRs 6*), *uracil-DNA glycosylase*, and a *disintegrin and metalloproteinase with thrombospondin motifs gon-1* (*ADAMTs*) genes (Table 4).

**Table 4:** The SNP markers, chromosome (Chr), MAF, and correlated genes from the GWAS analysis

Marker	Chr	Position	MAF	*P* value	Region	Adjacent Genes
AX_657645701	7	10,077,499	0.301902	1.98E-05	NA	*LOC123556079*, *LOC123555054*
AX_657591005	2	57,258,927	0.489699	3.70E-05	Exonic	*LOC123564010*
AX_657645697	7	10,077,291	0.291601	4.75E-05	NA	*LOC123556079*, *LOC123555054*
AX_657547767	5	57,690,595	0.200475	1.03E-04	NA	
AX_657943446	17	52,169,290	0.266244	1.24E-04	NA	
AX_657567998	5	57,690,175	0.199683	1.50E-04	NA	
AX_657896797	15	47,855,466	0.201268	1.59E-04	Intronic	*LOC123552332*, *LOC123557818*, *LOC123543633*, *LOC123561385*
AX_657666050	7	66,144,000	0.23851	1.71E-04	NA	
AX_657474782	3	52,244,152	0.073693	1.95E-04	NA	
AX_657554472	5	75,358,629	0.446117	2.99E-04	Exonic	*LOC123557653*, *LOC123558538*, *LOC123558539*, *LOC123558537*
AX_657384477	1	42,464,735	0.083201	3.04E-04	NA	*LOC123545639*, *LOC123540837*, *LOC123540855*
AX_657787276	18	22,080,941	0.019017	3.10E-04	Exonic	*LOC128550832*, *LOC128550505*

## Discussion

As one of the essential environmental factors for aquaculture, the optimum temperature range of *M. mercenaria* was evaluated for different life stages. In laboratory conditions, broodstock would respond to temperature for spawning at 23-30.2℃ [45]. After spawning, fertilized eggs could develop and grow to metamorphosis between 18 to 30℃. Within this range, the growth rates were generally higher at high temperatures, and temperature changes of 1–2 ℃ did not significantly affect the growth rate [46]. Combined with salinity, the optimum temperature for larval growth was assessed as 22.5–25℃ in brackish water and 17.5–30℃ at higher salinities [47]. At the juvenile stage, grow-out seed (10–15 mm shell length) and pasta-size seed (25–30 mm shell length) could tolerate 32 °C for longer than 15 days with only 1% and 4% mortality at the optimum salinity [28]. Adult quahogs can tolerate temperatures from below-freezing to about 35℃ [48]. The burrowing activity was observed at 5.5–36.6℃ and was the highest between 21–31℃ [49]. The pumping rate for feeding was observed at 6–32℃ with a maximum between 24–26℃ [50]. The siphon extension was observed at 1–34℃ and was the highest between 11–22℃ [51]. In this study, the heat challenge was conducted at 1℃ per day from 24℃ until reaching 35℃, and this plan was made by considering the quahog temperature tolerance, the in situ highest water temperature (34.09 ℃) in the clam leases in Florida, and our pilot experiment. The heaviest mortality occurred at ∼ 24 h after reaching 35℃, and after the temperature was back to normal, further heavy mortality continued (29–36%, Table 2) as a post-heat challenge effect. To ensure sufficient survivors (∼ 50%) for further breeding, the heat challenge could be lowered to 34℃ and ended when ∼ 25–30% mortality is achieved. In other bivalves, GWAS analysis of heat resistance also employed an acute heat challenge, such as submerging in semi-lethal temperature (42℃) for 1 h, and back to the optimum temperature for oysters [52], or calculation of thermal tolerant indicator - Arrhenius break temperature using a non-invasive optical sensor to measure heart rate in bay scallops [53].

The genetic analyses across farms and heat stress phases provide important insights into the role of genetic diversity and structure in the resilience of *M. mercenaria* to heat stress. The observed differentiation between clams that survived and those that succumbed to heat stress suggests selective pressures favoring certain genetic backgrounds, especially in Farms 1 (CK) and 3 (UF), where genetic shifts across phases indicated that specific genetic profiles are associated with higher mortality under thermal stress. The FastStructure analysis highlights specific ancestral components related to survival outcomes, particularly in CK and UF clams, where distinct genetic traits were associated with higher mortality. This suggests that specific genetic backgrounds in these farms may confer vulnerability to heat stress. This pattern was consistent with measures of inbreeding and heterozygosity, showing that genetic shifts in farms 1 and 3 are associated with selective survival based on heat tolerance. These findings underscore the importance of genetic structure in shaping survival outcomes under environmental stress. Breeding programs aiming at improving resilience in *M. mercenaria* should focus on maintaining genetic diversity and selecting heat-tolerant profiles, especially given the complex genetic dynamics observed at each farm. By targeting genetic stability and diversity, future efforts can help establish more resilient clam populations capable of adapting to environmental challenges.

Heritability is a statistical measure of genetic variation that contributes to the phenotypic variation in a population [54]. The SNP-based heritability estimate of 0.68 for the time-to-death trait in this study was higher than other traits reported in other bivalves, such as 0.375 heritability for survival status in *Mulinia lateralis* [55], 0.16 ∼ 0.36 for survival status and 0.16 ∼ 0.21 survival time in *Crassostrea gigas* [56], 0.25 ∼ 0.37 heritability for *Ostreid herpesvirus* (OsHV-1) resistance and 0.14 ∼ 0.27 for *Vibriosis* resistance [57–60], and 0.4 ∼ 0.5 heritability for low salinity tolerance *C. gigas* [61, 62]. Higher heritability suggests that genetic factors play a significant role in determining this trait in the studied population, indicating that a large proportion of the variation in time-to-death among individuals can be attributed to genetic differences rather than environmental influences. Therefore, the survival trait against heat challenge could be a target candidate for selective breeding programs. However, the high SNP-based heritability also suggests a potential need for further investigation into the genetic architecture underlying this trait, as such high estimates may reflect strong selection pressures or distinct population structures that differ from other bivalves. SNP-based heritability explains the cumulative effect of all SNPs contributing to the overall variation in the trait and may be inflated due to different population structure, fixed effects, and different methods [63].

The genome-wide association study (GWAS) performed with 38.7 K markers identified multiple SNPs that showed significant associations with the time-to-death trait, using the chromosome suggestive threshold. The Manhattan plot (Fig. 6) reveals several loci across different chromosomes that may be linked to survival traits. SNPs with *P*-values below 10^− 3.5^ were selected for further analysis, focusing on those located within a 50Kbp window around the identified SNPs to link them with nearby candidate genes. In this study, the chromosome-suggestive threshold was applied to identify the key regions or QTLs on the genome associated with heat tolerance. This threshold allowed for the identification of key regions that potentially harbor genetic variants affecting survival time. Although the identified SNPs did not reach the genome-wide significance threshold often used in GWAS, the selection of suggestive SNPs at this *P*-value threshold provides a starting point for exploring the genetic regions that contribute to the trait. Mapping these regions and further functional validation of the identified SNPs would help to clarify their role in heat tolerance or survival under specific stress conditions. Additionally, the *P* values were annotated in Table 4 for the identified genes for future validation. In our previous work with transcriptome analysis in response to chronic heat in *M. mercenaria* [27], the upregulated genes were involved in chaperone-mediated protein folding and regulation of cell death pathways, while the downregulated genes were involved in mRNA processing and splicing pathways. Possibly, it would be worth revisiting transcriptome data with the whole genome [42, 64] and the microarray data to identify heat tolerance-related candidate genes.

The GWAS analysis in other bivalve species for the heat tolerance trait identified associated SNPs and correlated candidate genes (Table 5**).** In dwarf surf clams, the genes in the ETHR/EHF signaling pathway were identified as correlating with heat tolerance, and their expression was confirmed with qPCR in the survivors of heat challenge [55]. In bay scallops, one gene encoding PC4 is involved in repairing DNA damage and stabilizing genome function, and 14 other SNPs were found associated with heat tolerance [53]. In the hybrids of *C. angulata* and *C. gigas*, GWAS analysis identified 18 SNPs and 26 candidate genes associated with heat tolerance. The genes included temperature sensing, transcription factor, protein stabilization, and solute carrier family, involving several pathways such as Ca^2+^ influx, transcriptional signal regulation, protein degradation or folding, and transport of metabolites pyruvate, to regulate the thermal response of marine organisms [52].

**Table 5:** Summary of GWAS analysis in aquaculture bivalves for heat tolerance

Species	Bay Scallop *Argopecten irradians irradians*	Hybrids of *Crassostrea angulata × C. gigas*	Dwarf clam *Mulinia lateralis*	Northern quahogs *Mercenaria mercenaria*
Reference	[53]	[52]	[55]	Current study
Heat challenge	Heart rate for Arrhenius break temperature	Submerging at 42℃ for 2 h, then back to normal temperature	At 30℃ for 10 days, then back to 22℃ for recovery	At 1℃/day from 24 to 35℃ and stay 2 days, back 24℃
Genotyping	Gill. Sequencing using multi-isoRAD approach	Gill. Sequencing	Gill. Sequencing using the iso-RAD approach	Muscle/hemocytes. ∼66K SNP array
Associated SNP	14 SNPs	18 SNPs 26 genes	3 SNPs 10 genes	1 SNP
Major Genes	PC4 gene for repairing DNA	Ca^2+^ influx, transcriptional signal regulation, protein degradation, or folding	ETHR/EHF signaling pathway	See Table 4
Heritability	NA	NA	0.375 ± 0.127	0.680 ± 0.063

### Gene function and relevance to heat tolerance

From the list of genes located within 50 Kbp of significant SNPs, several genes stand out for their potential roles in stress response, which could impact time to death under high-temperature stress. The candidate genes included **(1) Toll-like Receptor 4 (TLR4) and Toll-like Receptor 6 (TLR6)**. The Toll-like receptor (TLR) pathway is a class of membrane-bound immune effectors that activate intracellular innate immune signaling cascades and is the largest and most diversified receptor in molluscan bivalves [65]. Endogenous stress signals, such as those arising from tissue damage during heat stress, could activate TLRs. Their role in recognizing and triggering immune responses makes them plausible candidates for influencing survival under stressful conditions [66–69], and the involvement of TLR genes in heat challenge was confirmed in *Haliotis discus hannai* [70] and bay scallops [71]. **(2) Serine/threonine-protein kinase 31-like gene**. This gene is one type of MAPK (mitogen-activated protein kinases) gene family that plays an important role in the cellular response to environmental stress, specifically heat stress in plants [72]. Protein kinases are known to play roles in the regulation of heat-shock proteins and subsequently help protect cells from the damaging effects of elevated temperatures [73]. In *M. mercenaria*, five MAPK kinase genes were identified and were ubiquitously expressed in all tissues, especially the ovary. Specifically, MAPKK7 showed an upregulation in heat and heat plus hypoxia stress [74]. In *Meretrix petechialis*, the p38 MAPK gene was reported to contain a conserved serine/threonine protein kinase catalytic domain and showed a relationship with immune response [75]. In razor clams, serine/threonine-protein kinase was found to correlate with the heat stress response [73].

## Conclusions

The heritability of 0.68 for the time-to-death trait indicates a strong genetic influence on heat tolerance, making this trait a valuable target for breeding programs for broodstock improvement. The GWAS identified several suggestive loci, and the analysis of genes located near these SNPs points to potential roles in immune response, cellular stress, and tissue repair. Toll-like receptors, serine/threonine kinases, metalloproteinases, and DNA repair enzymes stand out as key candidates that could influence survival under heat stress or other environmental challenges. Further validation of these genes and their associated SNPs will be critical in determining their functional relevance to heat tolerance and survival in shellfish populations.

## Data Availability

The datasets generated and/or analyzed during the current study are available in the NCBI GEO repository, with an accession number of GSE290453.
